# Social Support and a Sense of Purpose: The Role of Personal Growth Initiative and Academic Self-Efficacy

**DOI:** 10.3389/fpsyg.2021.788841

**Published:** 2022-01-13

**Authors:** Jingxue Cai, Rong Lian

**Affiliations:** ^1^School of Psychology, Fujian Normal University, Fuzhou, China; ^2^School of Education, Fujian Polytechnic Normal University, Fuqing, China

**Keywords:** social support, sense of purpose, personal growth initiative, academic self-efficacy, serial mediation model

## Abstract

**Objective:** Studies have consistently found a positive relationship between social support and a sense of purpose; however, less is known about the underlying mechanisms of this relationship. The present study bridges this gap by proposing and testing a path model illustrating the mediating effects of personal growth initiative and academic self-efficacy on the linkage between social support and a sense of purpose.

**Method:** A total of 2,085 Chinese college students completed the revised versions of the Social Support, Personal Growth Initiative, Academic Self-Efficacy, and Sense of Purpose Scales.

**Results:** The results show that social support, personal growth initiative, and academic self-efficacy were all significantly associated with a sense of purpose. As predicted, personal growth initiative and academic self-efficacy mediated the relationship between social support and a sense of purpose, respectively. The results also support the hypothesized serial mediating effect.

**Conclusion:** Individuals who feel more social support have a higher level of personal growth initiative, their academic self-efficacy is stronger, and their academic self-efficacy further enhances their sense of purpose. Additionally, comparisons among the three indirect effects indicated that the effect of personal growth initiative was significantly greater than those of the other two measures. Thus, it can be concluded that personal initiative plays a greater role in enhancing a sense of purpose. These findings not only help to understand how social support enhances the sense of purpose, but also provide insight into the underlying mechanism.

## Introduction

A sense of purpose reflects the degree of individual feeling regarding a particular aim (Ryff, 1989). It does not involve the specific content of the goal (Burrow et al., 2010; Pfund et al., 2020). It can permeate all aspects of life to help allocate cognitive resources, stimulate behavioral consistency, and help individuals organize daily behaviors (Chen et al., 2019). When faced with competitive decisions (e.g., should I continue playing games or take a break?), people with a higher sense of purpose experience less neurological conflict and are more likely to cognitively make healthy behavioral decisions (Kang et al., 2019). Research has found that people who report a higher sense of purpose have greater wellbeing (Ryff, 1989; Sumner et al., 2015) and improved general cognitive performance (Lewis et al., 2017), are more physically active (Rector et al., 2019), feel more academic passion (Sharma and Yukhymenko-Lescroart, 2018), demonstrate better academic performance (Yeager et al., 2014), and rarely feel bored or listless (Bronk, 2014). Sharma et al. (2017) believed that whether or not college students were aware of their life purpose was a potentially important predictor that could be used to assess the level of their sustained hard work in school. Therefore, awakening and enhancing college students’ sense of purpose can not only give them meaning and power, but also motivate them to study hard and make great achievements. From a personal perspective, factors affecting one’s sense of purpose include personality characteristics, self-identity, and health (Weston et al., 2020b). From an environmental perspective, they also include growth background, social support, and social culture (Bronk, 2014; Hill and Weston, 2019).

It is an important resource that affects college students’ performance in school and future development, and includes the care received from parents, professional guidance from teachers, and mutual help among schoolmates. One study found that social support and its utilization significantly negatively predicted college students’ learning burnout (Liao, 2010). Social support has also been found to positively correlate with the emotional, normative, and ideal commitment of college students’ professional commitment (Zhou et al., 2012). Encouragement and guidance from influential others also play a key role in enhancing an individual sense of purpose, self-confidence, and ability to plan for the future (Sharma and De Alba, 2018). It has been confirmed that individual social support positively predicts a sense of purpose (Weston et al., 2020b). However, *how* does social support affect that sense of purpose? What is the internal mechanism? Unfortunately, there is as yet no relevant research on this topic. The present study used Chinese college students as participants to explore the ways in which social support affects one’s sense of purpose. This research will not only enrich the existing body of theoretical work on the factors influencing a sense of purpose, but also provide effective suggestions for enhancing it in college students.

### The Relationship Between Social Support and a Sense of Purpose

Social support refers to material or psychological resources from individuals’ social networks that help them cope with challenges (Taylor et al., 2015). Social support mainly includes support received from family, teachers, and peers. The generation and development of an individual sense of purpose is related to family members, teachers, peers, and communities (Moran et al., 2013; Liang et al., 2017). Families provide emotional, cognitive, and material resources. Family support affects the development of a sense of purpose during childhood, and even throughout adolescence (Bronk, 2014). Friends are an important source for college students. As young people spend more time with their friends, friendship becomes more and more important, so the support from friends grows stronger (Moran et al., 2013). Wentzel et al. (2016) argued that teachers were the most direct factor affecting students’ learning. Teachers’ recognition and emotional support have an important impact on improving the level of motivation and academic achievement in college students (Pan, 2017).

Research has shown that it is more important to perceive social support from parents and teachers than it is to simply be connected to them (Bundick and Tirri, 2014). A cross-lagged study found that college students’ perceived social support could significantly predict their feelings of hope at a later time (Xiang et al., 2020). Therefore, if college students feel positive social support and are encouraged by it, it helps them form a stronger sense of purpose, formulate appropriate learning plans, study hard, and persevere (Bronk, 2014; Weston et al., 2020a). Based on this, the following hypothesis is put forward: the social support perceived by college students has a positive effect on their sense of purpose.

### The Mediating Role of Personal Growth Initiative

According to the theory of self-determination, individuals generally have three basic needs: autonomy, relationships, and ability (Deci and Ryan, 2000). The need for autonomy refers to the need for an individual to act in response to their own will and sense of self-determination, without being controlled by others (Deci and Ryan, 2000). Robitschek (1998) called the tendency to consciously and actively improve and perfect oneself one’s personal growth initiative. Personal growth initiative is a manifestation of the need for autonomy and a core element of self-development. Research has shown that it is significantly positively correlated with college students’ academic effort (Chang and Yang, 2016), career exploration, and professional identity (Robitschek and Cook, 1999). Compared with individuals with low personal growth initiative, individuals with higher levels have clearer goals and formulate specific life plans according to life stages, making them more likely to achieve their goals (Xu et al., 2019). Therefore, it can be inferred that personal growth initiative will affect college students’ sense of purpose.

Personal growth initiative includes four dimensions: preparation for change, planning, use of resources, and conscious behavior (Robitschek et al., 2012). Cross-cultural research on personal growth initiative between Chinese and American college students has found obvious differences in the four dimensions. Chinese college students scored higher in the use of resources, while American college students scored higher in preparation and planning for change (Chang et al., 2017). This difference shows that American college students are better at planning, while Chinese college students are more connected with the outside world and better at using social resources. At the same time, feeling social support from the people around them can improve an individual’s level of personal growth initiative (Sun et al., 2014). According to ecosystem theory, environmental factors generally play a role through individual internal factors (Bronfenbrenner, 1979), so the social support perceived by an individual can only play a positive role in promoting self-growth when that individual actively transforms it into energy. College students feel the encouragement of social support and take the initiative to set appropriate goals to promote self-growth. Therefore, in the present study, we propose that personal growth initiative mediates the impact of social support on a sense of purpose.

### The Mediating Role of Academic Self-Efficacy

Academic self-efficacy is the specific application of self-efficacy in the field of learning. It refers to students’ expectations and judgments about their ability to complete specific learning tasks (Bandura, 1986). Studies have shown that academic self-efficacy positively predicts students’ learning motivation and academic performance (Wang et al., 2016). Research has also shown that academic self-efficacy significantly positively predicts students’ ability to set learning goals, as well as their effort level and ability to persist (Pajares, 2009). Consistent with Bandura’s (1986) self-efficacy theory, Liang et al. (2017) emphasized that individuals need to perceive their ability to achieve goals in order to effectively implement them. College students with stronger academic self-efficacy tend to be more interested in their goals and maintain a stronger sense of commitment to them. Therefore, academic self-efficacy is an influential factor in individuals’ sense of purpose.

Students’ academic self-efficacy is affected by the expectations, guidance, and social support given to them by important others (Chu et al., 2021). A longitudinal study confirmed that teacher support could predict students’ academic self-efficacy (Jungert and Koestner, 2015). College students perceive a teacher’s positive emotional support, which can then promote their interest in learning and academic self-efficacy (Liu et al., 2018). Students with higher academic self-efficacy have higher expectations for the future, can more quickly recover from adversity, are more likely to pursue challenging tasks, and make a greater commitment to their goals (Park and Yun, 2018). Thus, in the current research, we assumed that social support would affect a sense of purpose through academic self-efficacy.

### The Relationship Between Personal Growth Initiative and Academic Self-Efficacy

People with higher personal growth initiative have long-term plans, are better at allocating resources to achieve established goals (Parker et al., 2010). They have proposed a model of a proactive motivation process and antecedents, which demonstrate that personal growth initiative can directly affect self-efficacy. The model is premised on environmental factors (e.g., social support) and individual differences (e.g., personal growth initiative) affecting the individual’s active motivation state (e.g., self-efficacy) and whether the individual actively sets and achieves goals.

Self-efficacy is a dynamic construct, which is expected to change with changes in the environment (Wood and Bandura, 1989), whereas personal growth initiative is a relatively stable motivational tendency (Parker, 1998). A cross-lagged study showed that early personal growth initiative has a greater predictive effect on later academic self-efficacy than the reverse effect of later academic self-efficacy, indicating that personal growth initiative has an impact on academic self-efficacy (Lin et al., 2014). Longitudinal research has shown that personal growth initiative significantly predicts the amount of change in college students’ academic self-efficacy (Wang et al., 2016). Based on this, we propose that personal growth initiative may play a role by academic self-efficacy in the impact of social support on a sense of purpose.

### The Present Study

The present study was designed to explore the mechanisms underlying social support and a sense of purpose in Chinese college students. Further, a serial mediation model was built to reveal the unique effects of mediators and compare the strengths of the three indirect effects. Thus, according to previous studies, three hypotheses were proposed, as follows:

Hypothesis 1. Social support, personal growth initiative, and academic self-efficacy are significantly positively correlated and significantly positively associated with a sense of purpose.

Hypothesis 2. The relationship between social support and a sense of purpose is mediated by personal growth initiative and academic self-efficacy.

Hypothesis 3. Personal growth initiative and academic self-efficacy play a serial mediating role between social support and a sense of purpose.

## Materials and Methods

### Participants and Procedure

*Via* convenient sampling methods, a total of 2,085 college students were recruited from seven universities in five provinces in China. Questionnaires were distributed among them, incomplete and missing questionnaires were eliminated, 1,912 valid questionnaires were returned, and the recovery rate was 91.70%. Among them, female accounted for 62.7% and male accounted for 37.3%. Freshmen, sophomores, juniors, and seniors accounted for 34.4, 30.7, 32.2, 20.4, and 13% of the total number, respectively. Literature and history, science and engineering, accounting for 44.8 and 55.2% of the total, respectively. Urban, town and rural areas accounted for 24.5, 25.6, and 49.8% of the total, respectively. They were 18–23 years old, with an average age of 19.32 (SD = 1.58). Participants completed the self-report questionnaire during class time, which was presided over by well-trained researchers. Before the participants took the questionnaire, they obtained informed consent and ensured the anonymity of the answers. The Academic Ethics Committee of XX Normal University approved this study.

### Measurements

#### Social Support

Social support was accessed by the Multidimensional Scale of Perceived Social Support (Zimet et al., 1988). We use the Chinese version of the scale for this study. It consists of 12 items, including family, friends, and important others (SO) subscales, each with 4 items. Because it was conveniently applicable to college students, SO was replaced with Teacher Support. Individuals responded on a 5-point Likert scale, ranging from 1 (strongly disagree) to 5 (strongly agree); the higher the score, the stronger the sense of social support. E.g., “My parents can concretely help me.” In previous studies, this scale has been used for Chinese college students with good reliability and validity (Ye et al., 2014). The Cronbach’s alpha of the scale in this study was 0.84.

#### Personal Growth Initiative

The Personal Growth Initiative Scale-II (Robitschek et al., 2012) consists of 16 items, with responses recorded on a 5-point Likert scale (1 = “strongly disagree” to 5 = “strongly agree”). E.g., “I actively study and work to perfect myself.” The higher the score is, the stronger the personal growth initiative. The Cronbach’s alpha of the scale for the current study was 0.92.

#### Academic Self-Efficacy

The Academic Self-Efficacy Scale (Liang, 2000) was used in the present study. It consists of 22 items, Likert’s 5-point scoring, “1” means strongly disagree, and “5” means strongly agree. For example, “I believe that I have the ability to make achievements in my studies.” Higher scores indicate a stronger academic self-efficacy. The Cronbach’s alpha of the scale for the present study was 0.87.

#### Sense of Purpose

The self-compiled Sense of Purpose Questionnaire was used in the present study, including three dimensions: purpose awareness, purpose recognition, and purpose strategy. The questionnaire consists of 14 questions, with responses recorded on a 5-point Likert scale, “1” means strongly disagree, and “5” means strongly agree. The higher the score, the stronger the sense of purpose. E.g., “My purpose of life is clear.” The Cronbach’s alpha of the scale for the current study was 0.88.

## Results

### Preliminary Analyses

In this study, measures such as anonymous measurement and reverse scoring of some items were used to control the bias effect of the common method procedurally. Before analyzing valid data, we used the Harman single factor test method (Zhou and Long, 2004), there are 16 factors with eigenvalues greater than 1 when unrotated, explaining 58.38% of the variation. The first factor explains 21.93% of the variation, which is less than 40%, indicating that there is no serious common method bias problem.

Firstly, SPSS 25.0 was used for descriptive statistics and Pearson correlation analysis, to investigate the intercorrelations among social support, personal growth initiative, academic self-efficacy, and sense of purpose. Then, Hayes’s (2013) SPSS macro-PROCESS Model 6 with a 95% bias-corrected confidence interval (CI) based on 5,000 bootstrap samples was used to examine the serial intermediary variables in the complex models.

The normality assumption was checked after removing outliers, with all variables approximating a normal distribution. The single-sample *T* test showed that the total score range of college students’ sense of purpose is 3.48 ± 0.51, which was significantly different from the middle score of 3 (*t* = 40.87, *p* < 0.001), indicating that the overall level of college students’ sense of purpose was relatively high. College students’ sense of purpose had no significant differences in gender, major, and grade. There were significant differences in the scores of sense of purpose among college students from different places. The sense of purpose of urban college students was significantly higher than that of rural college students (*F* = 3.46, *p* < 0.05).

Table 1 presents the descriptive statistics and correlation matrix. Social support, personal growth initiative, and academic self-efficacy were significantly positively correlated, and the three significantly positively correlated with a sense of purpose. Thus, Hypothesis 1 was supported.

**TABLE 1:** Descriptive statistics and intercorrelations among variables (*n* = 1,912).

Variables	*M*	SD	Skewness	Kurtosis	1	2	3
(1) Social support	3.36	0.52	−0.22	0.16			
(2) Personal growth initiative	3.58	0.51	−0.28	0.31	0.55***		
(3) Academic self-efficacy	3.27	0.46	−0.80	−0.07	0.48***	0.67***	
(4) Sense of purpose	3.48	0.51	−0.29	0.15	0.46***	0.73***	0.60***

*** p < 0.001.

### Mediational Analysis

**FIGURE 1:**
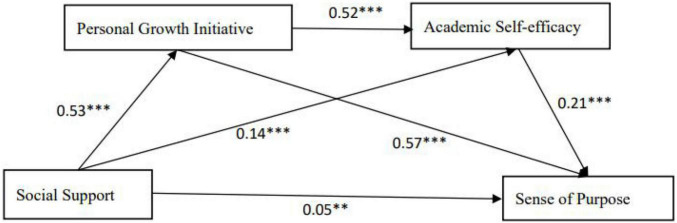
Serial mediation model showing the effects of social support, personal growth initiative, and academic self-efficacy on a sense of purpose. Values shown are unstandardized coefficients. ^**^p < 0.01, ^***^ *p* < 0.001.

The results (see Figure 1 and Table 2) show that social support significantly predicted personal growth initiative (β = 0.53, *p* < 0.001) and academic self-efficacy (β = 0.14, *p* < 0.001). In addition, social support, personal growth initiative, and academic self-efficacy all positively predicted a sense of purpose (β = 0.05, *p* < 0.001; β = 0.57, *p* < 0.001; β = 0.21, *p* < 0.001). Moreover, personal growth initiative positively predicted academic self-efficacy (β = 0.52, *p* < 0.001).

**TABLE 2:** Regression analysis among variables.

Regression equation		Global fit index			Significance of regression coefficient			
Outcome variable	Predictor variable	*R*	*R* ^2^	*F*	β	BootLLCI	BootULCI	*t*
Personal growth initiative		0.55	0.30	163.16				
	Social support				0.53	0.49	0.57	28.29***
Academic self-efficacy		0.68	0.47	278.82				
	Social support				0.14	0.11	0.18	8.25***
	Personal growth initiative				0.52	0.48	0.55	28.77***
Sense of purpose		0.74	0.55	338.49				
	Social support				0.05	0.01	0.09	2.73**
	Personal growth initiative				0.57	0.53	0.62	26.04***
	Academic self-efficacy				0.21	0.16	0.25	8.91***

Gender is the control variable. **p < 0.05, ***p < 0.001.

Table 3 presents the mediating effect between the variables. The 95% CI values of the three mediation paths did not contain zero, indicating that the mediation effect of the three paths was significant. Both personal growth initiative and academic self-efficacy were found to mediate the association between social support and sense of purpose, respectively. β = 0.30, SE = 0.02, 95% CI = [0.27, 0.34] for personal growth initiative and β = 0.03, SE = 0.01, 95% CI = [0.02, 0.04] for academic self-efficacy. It was also found that social support enhanced a sense of purpose through personal growth initiative and academic self-efficacy (i.e., a serial mediating effect), β = 0.06, SE = 0.02, 95% CI = [0.04, 0.07]. Thus, Hypotheses 2 and 3 were supported. Moreover, the sum of all indirect effects was 0.39 (95% CI = [0.36, 0.43]). The total relative mediation effect and effects of the three mediation paths were 88.64, 68.18, 6.82, and 13.64%, respectively.

**TABLE 3:** Mediating effects for variables.

Intermediary path	β	Boot SE	BootLLCI	BootULCI	Relative mediation effect
Total	0.39	0.02	0.36	0.43	88.64%
Ind1	0.30	0.02	0.27	0.34	68.18%
Ind2	0.03	0.01	0.02	0.04	6.82%
Ind3	0.06	0.02	0.04	0.07	13.64%

*N = 1,912. Unstandardized regression coefficients were reported. Bootstrap sample size = 5,000. LL, low limit, CI, confidence interval, and UL, upper limit. Ind1: social support→personal growth initiative→sense of purpose; Ind2: social support →academic self-efficacy→sense of purpose; and Ind3: social support→personal growth initiative→academic self-efficacy→sense of purpose.*

We conducted a pairwise comparison of the three indirect effects. The results indicate that the indirect effect of social support on a sense of purpose through personal growth initiative was significantly greater than the serial mediating effect, β = 0.27, SE = 0.02, 95% CI = [0.23, 0.31], and the indirect effect through academic self-efficacy, β = 0.25, SE = 0.02, 95% CI = [0.20, 0.29]. The serial mediation effect was stronger than the mediation effect of academic self-efficacy, β = −0.03, SE = 0.01, 95% CI = [−0.04, −0.01].

## Discussion

This research investigated the influence of social support on college students’ sense of purpose, as well as the mediating mechanism of personal growth initiative and academic self-efficacy from the self-determination theory, self-efficacy theory, and the Model of Proactive Motivation Process. The results show that the direct effect of social support on college students’ sense of purpose was significant (β = 0.44, *p* < 0.001). This study is consistent with the results of previous research; that is, a sense of purpose was found to be significantly related to the influence of important others (Moran et al., 2013; Weston et al., 2020a). After inputting the two intermediary variables of personal growth initiative and academic self-efficacy, the direct effect of social support on a sense of purpose was weakened (β = 0.05, *p* < 0.01). This showed that personal growth initiative and academic self-efficacy played a partially mediating role in the relationship between social support and a sense of purpose.

As with most psychological constructs, purpose does not develop in a vacuum; purposes are discovered, fostered, pursued, and realized with the support and guidance of friends, parents, and teachers and a variety of activities (Moran et al., 2013). According to Damon (2008), a sense of purpose could be inspired by teachers, strengthened by school activities, or supported by any family or friend who knows and understands the individual. Social support is perceived and comprehended by college students as a positive external resource; if inner needs are not awakened, the impact of social support may only be short-lived. Self-determination theory argues that intrinsic needs provide powerful motivation for individual goal-setting (Deci and Ryan, 2000), and personal growth initiative as an intrinsic and autonomous need provides intense energy useful for enhancing one’s sense of purpose (Ingrid et al., 2020). Universities essentially provide students with growth opportunities (Arnett, 2016). College students with high personal growth initiative are more likely to take advantage of this opportunity. Positive social recognition may enhance an individual’s personal growth initiative level, thereby enhancing their sense of purpose.

Specifically, it was found that the indirect effect of social support on a sense of purpose through academic self-efficacy was weaker than personal growth initiative. Perhaps university courses are relatively difficult and there is a lack of necessary communication between teachers and students, so the intermediary effect of academic self-efficacy is weaker. In any case, for many confused students, care and encouragement from people around them are vital to making them believe that they have the ability to complete their studies, maintain an appropriate sense of purpose, and actively participate in their education. However, as the saying goes, “you can never wake up a person who pretends to be asleep.” Therefore, only when based on the positions of “I am willing” and “I can do” will social support promote future planning and a general orientation toward the future. That is the main function of personal growth initiative and academic self-efficacy as chain intermediaries.

There are at least three limitations that should be considered. First of all, the data were collected by undergraduate self-reports, and the findings are prone to mono-method bias. Participants may not always accurately report their perceptions of social support, personal growth initiative, academic self-efficacy, and a sense of purpose, though the four scales were very internally consistent. Therefore, it is necessary for future research to confirm the findings by collecting data from individuals’ parents or teachers. Second, the current study created the intermediary model using a cross-sectional design, but it is not adequate to draw conclusions of any causality among the variables of social support, personal growth initiative, academic self-efficacy, and a sense of purpose (though the results suggest the possibility of such a causal link). Future work could be verified by longitudinal research and an experimental design. Third, the current study investigated the impact of social support on a sense of purpose, ignoring whether support coming from parents, teachers, or friends might have a stronger influence. Previous work has documented that teachers can foster a sense of purpose in their students in their mid to late adolescence (Bundick and Tirri, 2014). Therefore, future studies should consider distinguishing the influence of support from family and friends on a sense of purpose, and compare whose support is stronger in the same groups.

## Conclusion

This study found that a high level of social support perception could increase a sense of purpose, reminding educators and parents to intervene in college students’ sense of purpose, especially when guiding goal setting and goal pursuit. Furthermore, the present study found that personal growth initiative and academic self-efficacy can enhance the relationship between social support and a sense of purpose. In particular, the current study found that personal growth initiative had a stronger impact than academic self-efficacy on the linkage. Therefore, it is important to strengthen college students’ personal growth initiative and academic self-efficacy, especially to enhance their initiative. For instance, educators should stimulate students’ interest in learning, set challenging tasks, and give evaluative feedback, all of which should promote college students’ academic confidence and initiative, further enhance the sense of purpose of college students.

## Data Availability Statement

The data analyzed in this study is subject to the following licenses/restrictions: This research is a part of the first author’s Ph.D. thesis, the datasets generated and analyzed during the current study are not publicly available, because she is preparing her doctoral dissertation, but is available from the corresponding author on reasonable request. Requests to access these datasets should be directed to RL, lrong1122@126.com.

## Ethics Statement

The studies involving human participants were reviewed and approved by the Academic Ethics Committee of Fujian Normal University, China. The patients/participants provided their written informed consent to participate in this study. Written informed consent was obtained from the individual(s) for the publication of any potentially identifiable images or data included in this article.

## Author Contributions

RL proposed the research framework. JC designed the entire process, distributed and collected questionnaires, and analyzed the datas. Both authors participated in the compilation and verification of the manuscript, and approved the final manuscript.

## Conflict of Interest

The authors declare that the research was conducted in the absence of any commercial or financial relationships that could be construed as a potential conflict of interest.

## Publisher’s Note

All claims expressed in this article are solely those of the authors and do not necessarily represent those of their affiliated organizations, or those of the publisher, the editors and the reviewers. Any product that may be evaluated in this article, or claim that may be made by its manufacturer, is not guaranteed or endorsed by the publisher.
